# Cyclophosphamide augments the efficacy of *in situ* vaccination in a mouse melanoma model

**DOI:** 10.3389/fonc.2023.1200436

**Published:** 2023-09-06

**Authors:** Noah Tsarovsky, Mildred Felder, Mackenzie Heck, Jacob Slowinski, Kayla Rasmussen, Sabrina VandenHeuvel, Jen Zaborek, Zachary S. Morris, Amy K. Erbe, Paul M. Sondel, Alexander L. Rakhmilevich

**Affiliations:** ^1^Department of Human Oncology, Madison, WI, United States; ^2^Department of Biostatistics and Medical Informatics, Madison, WI, United States; ^3^Paul P. Carbone Comprehensive Cancer Center, Madison, WI, United States; ^4^Department of Pediatrics, University of Wisconsin, Madison, WI, United States

**Keywords:** melanoma, cyclophosphamide, radiation, immunocytokine, *in situ* vaccine.

## Abstract

**Introduction:**

We have previously shown that an intratumoral (IT) injection of the hu14.18-IL2 immunocytokine (IC), an anti-GD2 antibody linked to interleukin 2, can serve as an *in situ* vaccine and synergize with local radiotherapy (RT) to induce T cell-mediated antitumor effects. We hypothesized that cyclophosphamide (CY), a chemotherapeutic agent capable of depleting T regulatory cells (Tregs), would augment *in situ* vaccination. GD2^+^ B78 mouse melanoma cells were injected intradermally in syngeneic C57BL/6 mice.

**Methods:**

Treatments with RT (12Gy) and/or CY (100 mg/kg i.p.) started when tumors reached 100-300 mm^3^ (day 0 of treatment), followed by five daily injections of IT-IC (25 mcg) on days 5-9. Tumor growth and survival were followed. In addition, tumors were analyzed by flow cytometry.

**Results:**

Similar to RT, CY enhanced the antitumor effect of IC. The strongest antitumor effect was achieved when CY, RT and IC were combined, as compared to combinations of IC+RT or IC+CY. Flow cytometric analyses showed that the combined treatment with CY, RT and IC decreased Tregs and increased the ratio of CD8+ cells/Tregs within the tumors. Moreover, in mice bearing two separate tumors, the combination of RT and IT-IC delivered to one tumor, together with systemic CY, led to a systemic antitumor effect detected as shrinkage of the tumor not treated directly with RT and IT-IC. Cured mice developed immunological memory as they were able to reject B78 tumor rechallenge.

**Conclusion:**

Taken together, these preclinical results show that CY can augment the antitumor efficacy of IT- IC, given alone or in combination with local RT, suggesting potential benefit in clinical testing of these combinations.

## Introduction

During the last decade, an increasing number of immunotherapy approaches have shown clinical benefit for many patients with certain forms of cancer. Many preclinical studies have established that cancer treatment is most effective when several different therapeutic modalities are combined (1–4). We have previously shown that combining local radiotherapy (RT) with immunotherapy can be synergistic in several mouse tumor models (5). Combination of local RT and intratumoral immunocytokine (IT-IC), consisting of anti-GD2 antibody linked to interleukin 2 (IL-2), achieved an *in situ* vaccine effect, where immunomodulating treatments of the tumor induce activation of local and then systemic T cell responses against tumor antigens. The combination of RT and IT-IC resulted in regression of small GD2+ B78 melanomas in mice (5). However, regression of larger primary B78 tumors by this RT and IT-IC regimen, as well as regression of distant untreated tumors in these mice, required transient depletion of pre-existing, immunosuppressive, tumor infiltrating T regulatory cells (Tregs), either by using a Treg-depleting anti-CTLA-4 mAb or by testing in transgenic DEREG mice (6). Since IL-2 induces proliferation of Tregs, the blockade of Treg function may be required for this form of IC to have broader *in vivo* applicability (7). Therefore, testing of clinically relevant therapeutic strategies aiming at depleting Tregs in combination with this *in situ* vaccine is warranted.

Cyclophosphamide (CY) is an alkylating chemotherapeutic agent which inhibits tumor cell division by crosslinking DNA. It is widely used clinically for cancer therapy, including in combination with immunotherapy (8–10). Preclinical studies showed that CY can facilitate immunotherapy either in a T cell-independent manner (11) or in a T cell dependent manner by depleting Tregs (12, 13). Based on this well-known ability of CY to deplete Tregs, we hypothesized that CY may be beneficial for *in situ* vaccination either by replacing RT when combined with IT-IC or by augmenting the antitumor effects of a combination of RT and IT-IC.

## Materials and methods

### Mice

Female 7–8-week-old C57BL/6 mice were obtained from Taconic Farms (TAC, Germantown, NY). Mice were housed at the University of Wisconsin Biomedical Research Model Services vivarium in accordance with the Guide for Care and Use of Laboratory Mice. All experiments were performed under a protocol approved by the institutional Animal Care and Use Committee.

### Cell lines

GD2^+^ B78-D14 (B78) melanoma cells were obtained from Ralph Reisfeld (Scripps Research Institute) in 2002. They were derived from B78-H1 cells, which were originally derived from B16 melanoma. B78 tumor cells were grown in RPMI-1640 (Corning) supplemented with 10% heat inactivated FBS (Gibco), 2mmol/L L-Glutamine (Corning), 100 U/mL penicillin (Corning), and 100ug/mL streptomycin (Corning), with periodic supplementation with 400 μg/mL G418 and 50 μg/mL Hygromycin B. Mycoplasma testing by PCR was done routinely.

### *In vivo* murine models and treatments

B78 cells (2x10^6^) were injected intradermally (i.d.) as we previously reported (14) into the shaved right flank of syngeneic C57BL/6 mice (Taconic Farms). In the two-tumor model, mice were injected with B78 cells into both right and left flanks. Tumors were measured twice per week using electronic calipers. Tumor volumes were calculated using the formula: 0.5 x(small diameter)^2^ x large diameter. Mice were euthanized once their largest tumor diameter reached 20mm. Treatment began once tumors reached ~100-300mm^3^. Mice were dosed using an X-Rad 320 Animal Irradiator (Precision X-Ray). Mice received one fraction of radiation totaling 12Gy to their right flank tumors. The day of RT was day 0 of treatment for all experiments. Mice were injected intraperitoneally with CY at a dose of 100mg/kg on the day of RT. Mice were injected IT into the right tumor with hu14.18-IL-2 immunocytokine (IC, 50 mcg) (AnYxis Immuno-Oncology GmbH of Vienna, Austria) on days 5-9 following RT. In previous experiments (5), IT injection of rat IgG as a control for hu14.18-IL-2 IC did not cause tumor necrosis or antitumor effects; therefore, in this current study, control mice did not receive IT injections of IgG or vehicle (PBS). In rechallenge experiments, tumor-free or naïve mice were injected i.d. in the middle of the abdomen with 2x10^6^ B78 cells. We used the same dose of B78 tumor cell rechallenge as we used for initial tumor implantation, because we have established in previous experiments that mice cured by a combination of RT and IT-IC reject this dose of B78 tumor rechallenge while naïve mice do not (5).

### Flow cytometry

B78 tumor-bearing mice were randomized into control or treatment groups. On the indicated day after the treatments, mice were euthanized, and tumors were excised, minced, and digested into single cell suspensions using a Miltenyi gentleMACS Octo Dissociator (2.5 mL of complete RPMI media, 2.5 mg of Collagenase IV, and 250 g of DNase I per tumor). After dissociation, the resulting suspensions were passed through a 70m filter. Cells were then washed in PBS and incubated with Tonbo Ghost Dye Violet 510 for 30 min at 4°C, followed by washing in flow buffer (PBS-1% FBS) and a 10-minute incubation with anti-CD16/32 FC Shield (Tonbo, Clone 2.4G2) at 4°C. Cells were surface stained in Brilliant Stain Buffer (BD Biosciences) for 30 minutes at 4°C with CD45 FITC (30-F11), CD25 BB700 (PC61), CD8a APC-Fire 750 (53-6.7), and CD4 BV785 (GK1.5); the antibodies were obtained from BD Biosciences, BioLegend, or Invitrogen. Samples were washed, then fixed and permeabilized for 30 minutes at 4°C with the FoxP3/Transcription Factor Staining Buffer Set from eBioscience following kit instructions. Samples were then stained with FoxP3 PE-Cy7 (FJK-16s) from eBioscience for 30 minutes at 4°C. All samples were then washed in a permeabilization buffer and resuspended in flow buffer for acquisition on an Attune flow cytometer (ThermoFisher). Data were analyzed with FlowJo v10 software (BD). Tregs were defined as live, single, CD45+ CD4+ CD25+ FoxP3+ cells.

### Statistical methods

Tumor growth results from each mouse were summarized by the time-weighted average (area under the volume-time curve, calculated using trapezoidal method). Time-weighted averages were compared between treatment groups overall by Kruskal-Wallis tests. If significance was found using the Kruskal-Wallis test, then pairwise comparisons were conducted using Mann-Whitney tests. No p-value corrections were applied to the pairwise tests. Survival curves were compared with log rank tests.

## Results

### Antitumor effects of CY in combination with IC or RT

**Figure 1:**
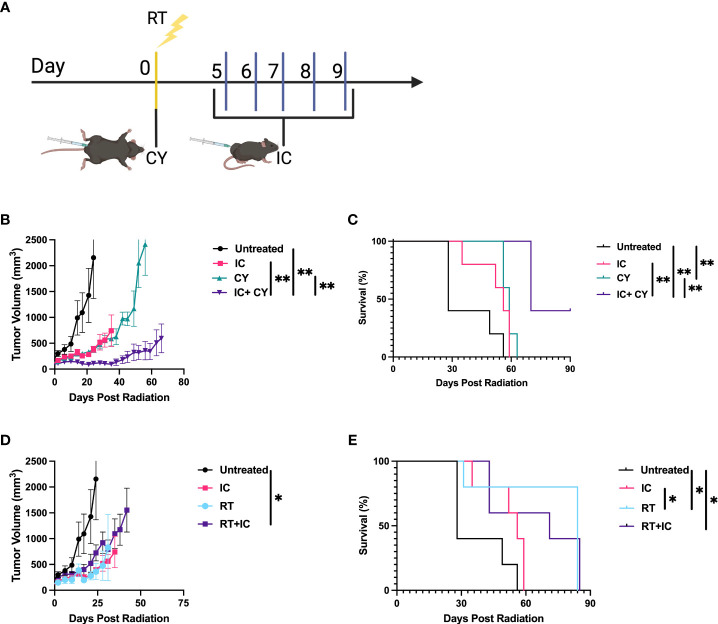
CY and RT improve the antitumor efficacy of IC. **(A)** Schema of dosing schedule for RT, IC, and CY. Treatment began when the tumor reached a mean volume of ~100-300mm^3^. **(B, C)** compare IC, CY and IC+CY; **(D, E)** compare RT, IC and RT+IC. Mean +/- SEM of the tumor volumes **(B, D)** and mouse survival **(C, E)** are shown (n=5). The results for each pair of tumor growth and survival data **(B–E)** are representative of two independent experiments. *p≤ 0.05; **p≤ 0.01. p values comparing all groups to one another are included in **Supplemental Table 1**.

We hypothesized that CY will be beneficial when combined with IC or RT+IC because it was reported that CY can improve immunotherapy by reducing Tregs (12, 13). Therefore, first we wanted to confirm that CY reduces Tregs within the tumors in our B78 melanoma model. **Supplemental Figure 1** shows that indeed, Tregs were significantly reduced 5 days after a single injection of CY. Next, we tested if CY would augment the antitumor effect when combined with IC. The treatment schema is depicted in **Figure 1A**. When CY was combined with IT-IC, a significant reduction of tumor growth (**Figure 1B**) and prolongation of survival compared to untreated mice (**Figure 1C**) were achieved, whereas treatment with IT-IC alone or CY alone did not show statistically significant tumor reduction. In addition, the combination of CY and IT-IC had significantly greater effect than CY or IT-IC in both suppressing tumor growth and extending the survival. The combination of IC and RT, in contrast to RT or IC alone, significantly reduced growth of the tumors that were ~150mm^3^ when treatment began (**Figure 1D**). This combination of RT and IC also prolonged survival compared to untreated mice, although RT alone prolonged survival as well (**Figure 1E**). These findings confirm the beneficial effect of combining RT and IT-IC, as we reported previously (5).

### Antitumor efficacy of a triple combination of CY, IT-IC and RT

**Figure 2:**
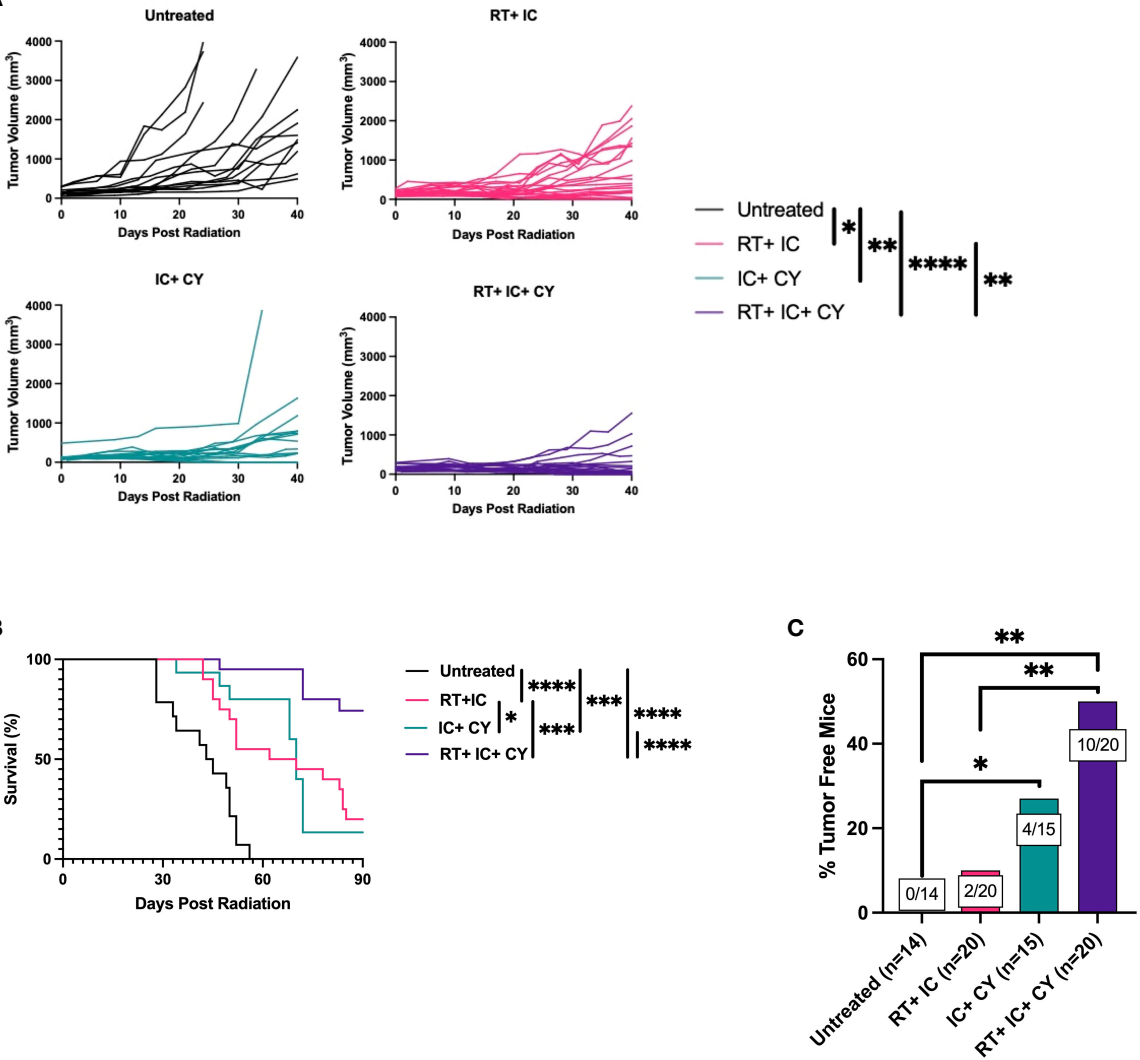
A combination of CY, RT and IT-IC results in enhanced antitumor efficacy against B78 melanoma. Mean +/- SEM of tumor volumes **(A)** and mouse survival until day 90 post RT **(B)** are shown. The percentages of tumor-free mice until day 175 post RT are shown. The ratio on each bar shows the number of tumor-free mice out of the total number of mice from four independent experiments **(C)**. The results show the combined data of four independent experiments with p-values from proportion tests. *p≤ 0.05; **p≤ 0.01;***p ≤ 0.001; ****p ≤ 0.0001.

**Figure 3:**
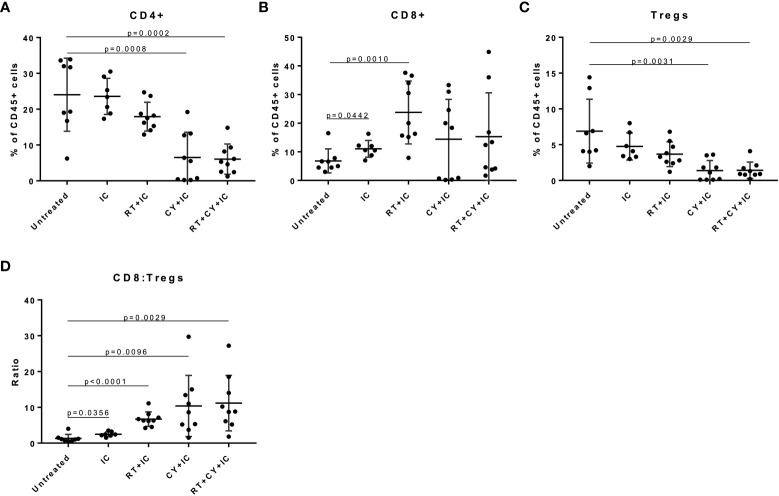
Treatments with CY in combination with IC or RT+IC induce reduction of Tregs. B78 tumor-bearing mice were treated with RT and/or CY on Day 0 and/or IC on Day 5-9 as shown in **Figure 1A**. Flow cytometry of tumor-infiltrating lymphocytes was performed on day 10. Percentages of CD4^+^ T cells **(A)**, CD8^+^ T cells **(B)**, Tregs **(C)** and ratio of CD8^+^ T cells/Tregs **(D)** within the population of CD45^+^ cells are shown and were compared using unpaired t-test. The combined data from two independent experiments are shown (n=9 mice per group).

Next, we asked if combining all three treatment modalities, RT, immunotherapy (IT-IC) and chemotherapy (CY), would be more effective than IT-IC combined with RT or with CY. **Figure 2** shows the combined data of four separate experiments. When CY was combined with RT and IT-IC, this triple modality combination induced a statistically significant reduction of tumor growth compared with untreated mice and with mice treated with RT + IC, whereas this effect for the triple modality was not statistically significant from IC + CY (**Figure 2A**). However, this combination of RT, IC and CY induced statistically significant prolongation of survival of mice compared to RT + IC or IC + CY (**Figure 2B**). No treatment-related toxicities (ruffled fur, lethargy or diarrhea) were observed. In addition, the combined treatment with RT, IC and CY showed a greater number and percentage of cured mice (10/20) than in RT + IC (2/20) or IC + CY (4/15) treated groups (**Figure 2C**), although the difference between the triple group and IC+CY was not statistically significant. Overall, the results in **Figure 2** show the advantage of combining CY with RT and IT-IC. Flow cytometry analyses of tumor-infiltrating lymphocytes on day 10 (10 days after RT and CY injection and 1 day after the last treatment with IT-IC) showed that the mice receiving the triple combination of RT, CY and IC, as well as mice treated with CY+IC, had a reduced percentage of CD4^+^ T cells (**Figure 3A**) whereas the relative number of CD8^+^ cells was not changed (**Figure 3B**). Importantly, the mice receiving the triple combination of RT, CY and IC, as well as mice treated with CY+IC, had significantly reduced percentages of Tregs (**Figure 3C**) and increased ratios of CD8^+^ T cells/Tregs (**Figure 3D**) compared to untreated mice, suggesting greater activation of effector T cells. There were no reproducible statistical differences of macrophages and myeloid cells between the groups (data not shown).

### The triple combination of CY, IT-IC and RT induces a systemic antitumor response and immunological memory

**Figure 4:**
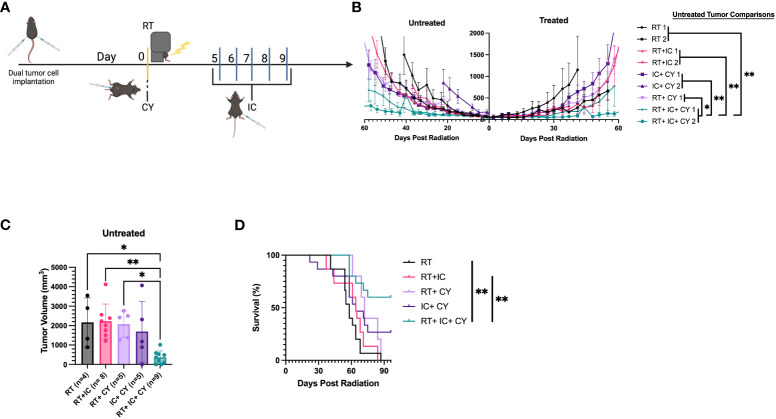
Combination of CY, RT and IT-IC induces systemic immunity against B78 Melanoma. **(A)** Schema of RT + IT-IC + CY treatment regimen. C57BL/6 mice were injected with B78 tumor cells on both the right and left flank. Right flank (treated) tumors were given RT on day 0 once their right flank tumor reached ~100mm^3^. CY was injected on Day 0. IC was injected IT into right tumors on days 5-9. **(B)** Data shown are Means +/- SEM of tumor volumes, for the indicated treatment groups. (n=5 per group per experiment). Statistical comparisons of the response at the untreated tumor are shown, for the data from two independent experiments (labelled 1 and 2 in the statistical comparison schematic), except for RT + CY group which was done in one experiment. **(C)** Tumor volumes (Mean +/- SEM) of the untreated tumor from mice bearing two B78 tumors on day 61 following RT were compared using one-way ANOVA. **(D)** Combined survival of mice bearing two B78 tumors from two independent experiments [n=10, except RT+CY(n=5)] is shown. *p≤ 0.05; **p≤ 0.01. P-values from the statistical comparisons between groups are included in **Supplementary Table 1**.

Next we tested if the triple combination of CY, RT and IT-IC induced a systemic antitumor immune response. B78 tumor cells were injected simultaneously in mice on both the right and left flanks. When the tumors reached ~ 100mm^3^, the tumors on the right were treated with RT and IT-IC (**Figure 4A**), while the CY was given i.p. When these mice were treated with the RT+IC+CY combination, antitumor effects were noted against the left (untreated) tumors (**Figure 4B**). When we compared the volumes of the untreated tumors of mice across two experiments, we found that 61 days following RT, mice treated with RT+ IC+ CY had significantly more tumor regression compared to mice treated with RT, RT+IC, or RT+CY (**Figure 4C**). Survival following the triple treatment was significantly improved as compared to RT and RT+IC (**Figure 4D**). The overall statistical differences between the groups, and between tumor volumes on day 61, are presented in the **Supplemental Table 1**. There was no statistical differences on day 61 between tumor volumes in untreated (**Figure 4C**; Mean +/- SD = 267 +/- 378, n=9) and treated (**Supplemental Figure 2**; Mean +/- SD=364 +/- 330, n=9) tumors following RT+IC+CY therapy, although the untreated tumor group had 2/9 mice with complete tumor regression, whereas the treated tumor group had 4/9 mice with complete tumor regression. Overall, the results with a two-tumor model show that a combination of RT+IC+CY can elicit a systemic antitumor response against a distant tumor.

**Figure 5:**
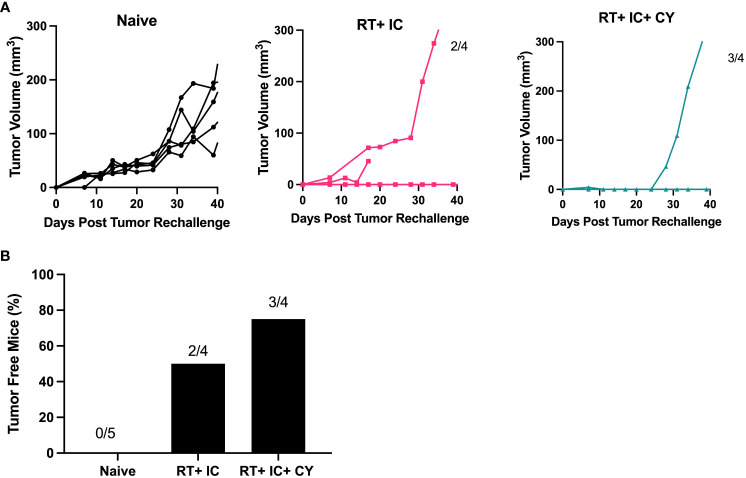
Combination of CY, RT and IC enables immune memory against B78 Melanoma. After mice had been tumor-free for at least 60 days following treatment-induced B78 single flank tumor rejection, these mice and naïve mice were injected with 2x10^6^ B78 melanoma cells on the abdomen. **(A)** Individual mouse tumor growth curves for naïve mice, or mice previously cured by RT+ IC or RT+ IC+ CY following implantation with B78 tumor cells are shown. The ratio above each graph indicates the number of tumor-free mice out of the total number of implanted mice. **(B)** Percentages of tumor-free mice, calculated from the number of tumor-free mice out of total number of mice as indicated above the bars in **(A)**, are shown.

To test if our treatments resulted in long term immunological memory, cured mice were rechallenged with B78 tumor cells 60 days after the primary tumor rejection. The results show that the mice that were cured by a combination of RT+IC or RT+ IC+ CY, in contrast to naïve mice, were able to reject rechallenge of B78 tumors (**Figures 5A, B**), indicating that they developed immunological memory to B78 melanoma.

## Discussion

Preclinical and clinical studies demonstrate that immunotherapy can be effective against certain cancers (14–18). The efficacy of many forms of immunotherapy can be enhanced by combining with conventional treatments such as RT and chemotherapy (19). Several preclinical studies have evaluated combinations of immunotherapy with either RT (4, 20) or chemotherapy (1, 8). However, few preclinical studies have evaluated combinations of all three therapeutic modalities: RT, chemotherapy, and immunotherapy (21, 22). In this study, we show that the triple combination of local immunotherapy (IT-IC), RT and chemotherapy (CY) is more effective against B78 melanoma than the combination of IT-IC with RT or CY.

We have previously shown that IT-IC can synergize with local RT to induce tumor regression in the murine B78 melanoma (5). B78 melanoma, which is derived from a male mouse, is immunologically cold even in female mice, in that it didn’t respond to the checkpoint inhibitor anti-CTLA-4 (5). The beneficial interaction of RT with IC involves a number of factors that include upregulated antigen expression on irradiated tumor cells, beneficial changes in the tumor micro-environment, greater susceptibility of tumor cells to immune mediated cell death signals (e.g., Fas ligand) and release of chemokines and cytokines that all facilitate more effective tumor recognition and destruction by T cells (5, 20). While RT and IT-IC induced regression of small B78 tumors, we showed that the antitumor efficacy of this treatment was inhibited in mice with a larger B78 tumor load due to the presence of Tregs (6). Therefore, we hypothesized that CY, a chemotherapeutic drug capable of depleting Tregs (12, 13), would augment the therapeutic effect of RT + IT-IC. Our results confirm this hypothesis by showing that CY can augment the antitumor efficacy of IT- IC in combination with local RT.

First, we confirmed that CY at the dose of 100 mg/kg depleted Tregs in our B78 melanoma model. Our results showing that Tregs were reduced in the tumors on day 5 after injection of CY (**Supplemental Figure 1**) are in keeping with the similar observations by Lutsiak et al. (13). This timing of Treg depletion after CY correlated with the enhanced antitumor effect when IT-IC is given 5 days after CY, suggesting that immunotherapy is more effective when it is administered at the time when Tregs are reduced or depleted. This confirms the similar observations of giving CY before immunotherapy to achieve better antitumor or immunostimulating efficacy (23), whereas CY given at the time of immunotherapy with systemic IL-2 led to enhanced tumor growth rather than tumor inhibition (24). Our findings that the combination of CY, RT, and IT-IC depleted Tregs in the TME on day 10 (**Figure 3**) are in agreement with the observation that another form of immunotherapy, anti-PD-1, prolonged Treg depletion by CY (25).

Our results showing that CY synergizes with IT-IC is consistent with prior reports from our team (11) and from others (8, 12) on the enhanced antitumor efficacy of CY combined with other forms of immunotherapy. Importantly, our results demonstrate that this triple combination of CY, RT and IT-IC induced a better antitumor effect resulting in more cures than a combination of IT-IC with RT or with CY. These results are consistent with other reports (21, 26) showing the enhanced antitumor effect of combining CY, local RT, and dendritic cell vaccine. The antitumor effect observed in our study was not limited to the tumor treated directly with IT-IC and RT, but it also reduced growth of a distant tumor (not receiving the IT-IC or RT), suggesting that this treatment strategy can be effective against metastatic tumors. Because the combination of CY, RT and IT-IC decreased the percentage of Tregs in the tumor and increased the ratio of CD8^+^ T cells/Tregs, we suggest that reduction of Tregs by CY at the time of immunotherapy and during the course of the treatment contributed to the enhanced antitumor effect. However, because **Figure 3** showed no significant differences in percentage of Tregs and CD8:Treg ratio between the triple combination (RT+IT+CY) and double combinations (RT+IC and CY+IC), it is possible that CY-induced reduction of angiogenesis (27), activation of innate immunity (28), or other mechanisms can contribute to the antitumor benefit of the triple combination.

One of limitation of this study is using only female mice; therefore, these findings need to be confirmed in male mice before proposing clinical use of this treatment combination. Another consideration is whether the 14.18-IL-2 IC can be replaced with IL-2 alone or IL-2 + anti-GD2 mAb, to make this approach more clinically applicable for various cancers including GD2 negative melanomas. We have found that indeed, in combination with RT, IT injection of IL-2 was effective against B78 melanoma, and this antitumor effect was slightly augmented by IT injection of anti-GD2 mAb (Jin W. et al., submitted). The role of antibody specificity for the treated tumor when using ICs is also relevant here. Prior studies have shown that mAb-IL-2 IC fusion proteins are effective against the B78 when given intravenously when the mAb component is an anti-GD2 mAb, but not when it is a separate mAb that recognizes EpCAM (and that is effective against EpCAM^+^ tumors); B78 is GD2^+^ but EpCAM^-^ (29). These studies clarify the importance of antibody specificity in the action of ICs.

Some recent clinical trials used triple combinations of systemic immunotherapy, RT and chemotherapy (30, 31) showing a therapeutic benefit. IT immunotherapy has been increasingly used as an *in situ* vaccination approach for treatment of certain cancers including melanoma (32). We are currently leading an ongoing trial of IT hu14.18-IL-2 in combination with local RT for advanced melanoma at our institution (NCT03958383). The results presented here show the beneficial use of the triple combination of RT, IT-IC and CY in the context of this *in situ* vaccine approach for B78 melanoma and suggest continued clinical testing of chemo-radio-immunotherapy.

## Data availability statement

The original contributions presented in the study are included in the article/**Supplementary Material**, further inquiries can be directed to the corresponding author/s.

## Ethics statement

The animal study was approved by UW Madison Institutional Animal Care and Use Committee (IACUC). The study was conducted in accordance with the local legislation and institutional requirements.

## Author contributions

NT, MF and AR were responsible for experimental design, execution, and analysis of data. NT and MF created final versions of all figures. NT and AR drafted the manuscript. NT, MF, MH, JS, KR, SV collected and analyzed experimental data. JZ conducted and confirmed statistical analysis of experimental data. PS contributed to experimental design and thorough editing of the manuscript, AR, AE, and ZM contributed to experimental design and review of the manuscript. All authors provided thorough review and editing of the manuscript draft. All authors contributed to the article and approved the submitted version.

## Supplementary material

Supplementary Figure 1 CY induces reduction of Tregs. B78 tumor-bearing mice were treated with CY, and flow cytometry of tumor-infiltrating lymphocytes was performed 5 days later. Percentages of CD4^+^ T cells **(A)**, CD8^+^ T cells **(B)**, Tregs **(C)** and ratio of CD8^+^ T cells/Tregs **(D)** within the CD45^+^ cell gate are shown (n=5). Dot plots of Tregs (CD25^+^/FoxP3^+^) within the CD45^+^ CD4^+^ gated cells are shown for representative individual untreated and CY-treated mice **(E)**. The results are representative of two independent experiments.

Supplementary Figure 2 Treated Tumor Volume Comparions of Dual Flank B78 melanoma model shown in **Figure 4**. Tumor volumes (Means +/- SEM) of the treated (right) tumors on day 61 of mice from the dual flank B78 model in . The differences between the groups were not significant. P-values are shown in **Supplemental Table 1C**.

Supplementary Table 1 P-values and mean tumor volumes from the statistical analysis of groups in **Figure 4** and **Supplemental Figure 2**. **(A)** p-values for untreated tumor volume comparisons across groups at all time points until day 100 (). **(B)** p-values for survival comparisons of mice in a two-tumor B78 model (). **(C)** p-values for one-way ANOVA analysis of treated tumor volumes on day 61 following RT (**Supplemental Figure 2**). **(D)** p-values for one-way ANOVA analysis of untreated tumors on day 61 following RT (). **(E)** Mean tumor volumes of treated (**Supplemental Figure 2**) and untreated tumors () for each treatment group at day 61 post RT. The p-values show statistical comparison of the combined data of two similar experiments [(n=10), except RT+CY (n=5)]. *p≤ 0.05; **p≤ 0.01.
