# Supplementary figures and images for: Cyclophosphamide augments the efficacy of in situ vaccination in a mouse melanoma model
Source: Front Oncol. 2023 Sep 6;13:1200436. doi: 10.3389/fonc.2023.1200436 (PMC10516537; doi:10.3389/fonc.2023.1200436)

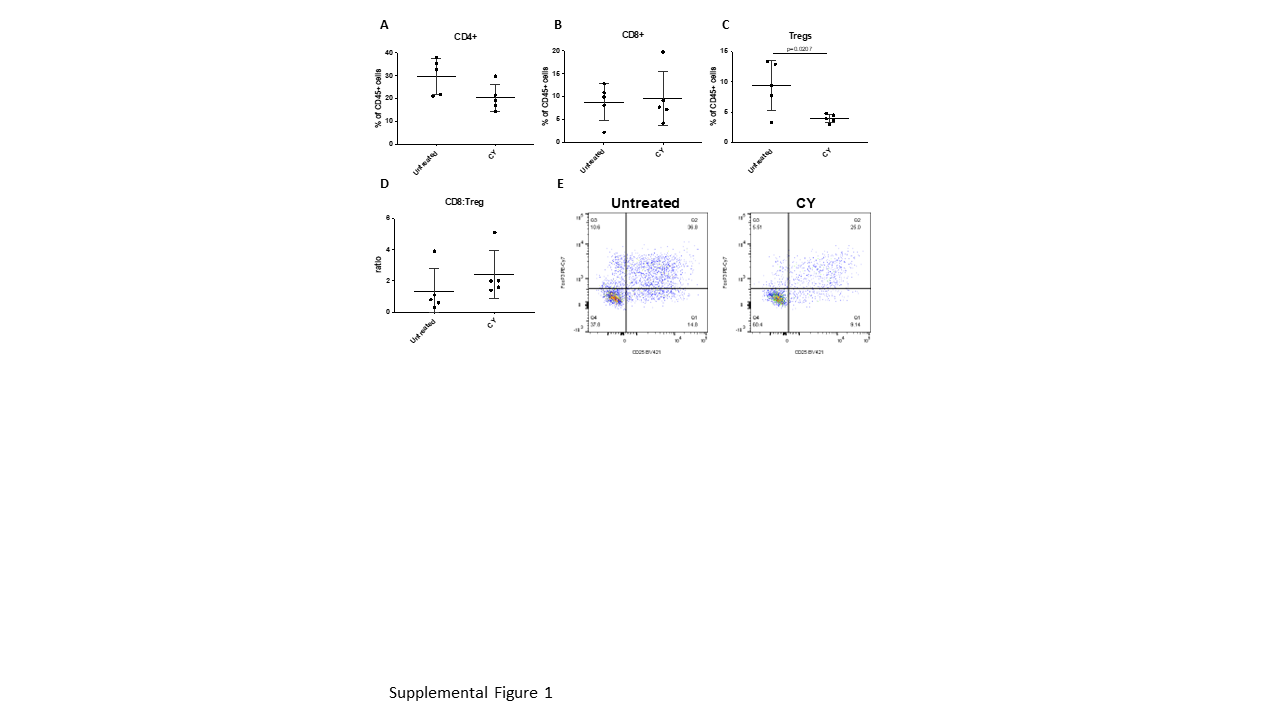

Supplement: Supplementary Figure 1 — CY induces reduction of Tregs. B78 tumor-bearing mice were treated with CY, and flow cytometry of tumor-infiltrating lymphocytes was performed 5 days later. Percentages of CD4+ T cells (A), CD8+ T cells (B), Tregs (C) and ratio of CD8+ T cells/Tregs (D) within the CD45+ cell gate are shown (n=5). Dot plots of Tregs (CD25+/FoxP3+) within the CD45+ CD4+ gated cells are shown for representative individual untreated and CY-treated mice (E). The results are representative of two independent experiments. [file Image_1.tif]

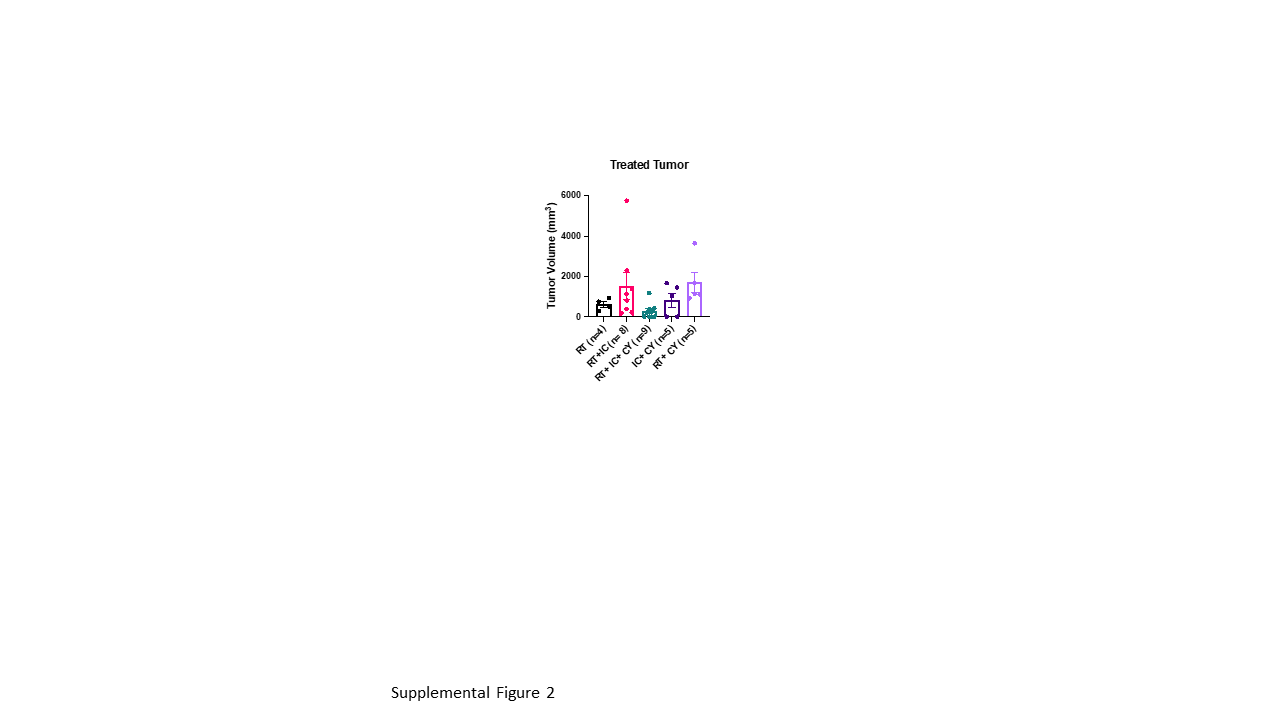

Supplement: Supplementary Figure 2 — Treated Tumor Volume Comparions of Dual Flank B78 melanoma model shown in Figure 4. Tumor volumes (Means +/- SEM) of the treated (right) tumors on day 61 of mice from the dual flank B78 model in . The differences between the groups were not significant. P-values are shown in Supplemental Table 1C. [file Image_2.tif]

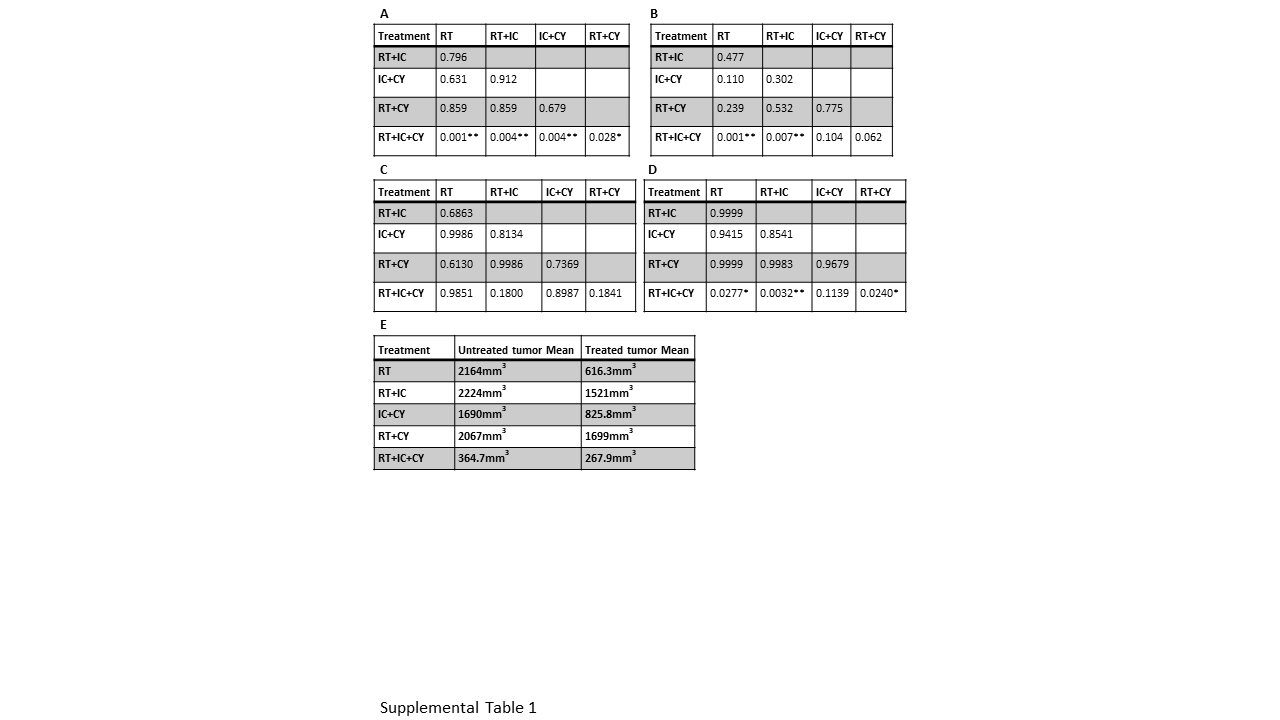

Supplement: Supplementary Table 1 — P-values and mean tumor volumes from the statistical analysis of groups in Figure 4 and Supplemental Figure 2. (A) p-values for untreated tumor volume comparisons across groups at all time points until day 100 (). (B) p-values for survival comparisons of mice in a two-tumor B78 model (). (C) p-values for one-way ANOVA analysis of treated tumor volumes on day 61 following RT (Supplemental Figure 2). (D) p-values for one-way ANOVA analysis of untreated tumors on day 61 following RT (). (E) Mean tumor volumes of treated (Supplemental Figure 2) and untreated tumors () for each treatment group at day 61 post RT. The p-values show statistical comparison of the combined data of two similar experiments [(n=10), except RT+CY (n=5)]. *p≤ 0.05; **p≤ 0.01. [file Image_3.tif]
